# The Good, the Bad, and the Ugly—Chances, Challenges, and Clinical Implications of Avoidance Research in Psychosomatic Medicine

**DOI:** 10.3389/fpsyt.2022.841734

**Published:** 2022-02-18

**Authors:** Franziska Labrenz, Marcella L. Woud, Sigrid Elsenbruch, Adriane Icenhour

**Affiliations:** ^1^Department of Medical Psychology and Medical Sociology, Ruhr University Bochum, Bochum, Germany; ^2^Mental Health Research and Treatment Center, Department of Psychology, Ruhr-University Bochum, Bochum, Germany; ^3^Department of Neurology and Center for Translational Neuro- and Behavioral Sciences, University Hospital Essen, Essen, Germany

**Keywords:** avoidance, psychosomatic medicine, pain, anxiety, associative learning, conditioning, gut-brain axis

## Abstract

Avoidance behaviors are shaped by associative learning processes in response to fear of impending threats, particularly physical harm. As part of a defensive repertoire, avoidance is highly adaptive in case of acute danger, serving a potent protective function. However, persistent or excessive fear and maladaptive avoidance are considered key factors in the etiology and pathophysiology of anxiety- and stress-related psychosomatic disorders. In these overlapping conditions, avoidance can increase the risk of mental comorbidities and interfere with the efficacy of cognitive behavioral treatment approaches built on fear extinction. Despite resurging interest in avoidance research also in the context of psychosomatic medicine, especially in conditions associated with pain, disturbed interoception, and disorders of the gut-brain axis, current study designs and their translation into the clinical context face significant challenges limiting both, the investigation of mechanisms involved in avoidance and the development of novel targeted treatment options. We herein selectively review the conceptual framework of learning and memory processes, emphasizing how classical and operant conditioning, fear extinction, and return of fear shape avoidance behaviors. We further discuss pathological avoidance and safety behaviors as hallmark features in psychosomatic diseases, with a focus on anxiety- and stress-related disorders. Aiming to emphasize chances of improved translational knowledge across clinical conditions, we further point out limitations in current experimental avoidance research. Based on these considerations, we propose means to improve existing avoidance paradigms to broaden our understanding of underlying mechanisms, moderators and mediators of avoidance, and to inspire tailored treatments for patients suffering from psychosomatic disorders.

## Introduction

Learning to flexibly respond to dynamic environmental challenges constitutes a highly adaptive mechanism aimed at self-protection, particularly when faced with impending physical harm (1). One of the most defensive, yet in case of acute threat particularly protective responses is avoidance behavior (2). Based on the influential fear avoidance model (FAM) (3, 4), which has initially provided a theoretical framework for the pathology of fear, avoidance behavior involves a cascade of fear-related responses shaped by associative learning, particularly classical and operant conditioning. While beneficial during phases of acute danger, when threats cease but fear and its consequences persist, avoidance or safety behaviors lose their adaptive function and the formerly protective cascade becomes maladaptive. This can initiate a vicious circle of fear and distress, potentially culminating in the development and persistence of disease (5, 6).

More recently, the FAM has been extended to conceptualize the key role of fear in chronic pain (7–10) and disturbances of the gut-brain axis (11) as highly common psychosomatic disorders (12–14) presenting with eminent comorbidity rates with anxiety disorders (15). Evidence from experimental research in these fields supports the assumptions of the FAM, showing that pain-related fear and avoidance are associated with dysfunctional cognitive, behavioral, and affective responses, including negative appraisal, catastrophizing, and hypervigilance (16–20). These factors promote the maintenance and exacerbation of symptoms and contribute to comorbid psychiatric disorders, increased distress, functional disability, social withdrawal, and reduced quality of life (21–24).

Avoidance has highly relevant treatment implications, providing a foundation for therapeutic interventions following associative learning principles (25–27), particularly exposure-based treatments based on fear extinction within the framework of cognitive behavioral therapy (CBT) (28–30). The main aim of exposure is to help patients to confront themselves to cues and contexts they fear and avoid, and to endure their fears and corresponding behavioral, emotional, and cognitive responses (31). Systematic exposure demonstrably reduces anxiety-relevant symptoms and improves functional abilities (30–33). According to recent findings, extinction effects are initiated by a violation of the patients' dysfunctional expectancies (34, 35). Therefore, during exposure, patients are often encouraged to refrain from engaging in avoidance and safety behaviors, providing an opportunity to experience fear-correcting situations (36–38).

Despite this outstanding relevance in the transition from acute threat to chronic disease, persistence of symptoms, and therapeutic interventions, for a long time avoidance has not received the attention owed in experimental research (39), particularly in the context of psychosomatic medicine. Attempts to investigate avoidance in experimental pain research have more recently been made (40, 41), and avoidance and safety behaviors have been identified as crucial mechanisms of action in CBT for patients suffering from disorders of gut-brain interaction (42, 43). However, experimental approaches often fall short with respect to validity criteria and do not adequately translate into clinical contexts (27, 44–47), and systematic empirical investigations in psychosomatic disorders are still widely lacking.

We herein selectively highlight experimental considerations and clinical implications of avoidance behaviors in the context of psychosomatic disorders with a particular focus on frequently co-occurring pain conditions, disorders of the gut-brain axis, and anxiety disorders (48). We discuss chances but also methodological and conceptual challenges in establishing clinically-relevant experimental models to elucidate avoidance behaviors, underlying mechanisms, and their role in long-term effects of CBT for anxiety and psychosomatic disorders.

## A Conceptual Framework of Learning and Extinction

Avoidance is embedded within a conceptual framework of associative learning governed by principles of classical and operant conditioning. Fear conditioning provides an excellent model for investigating the development and maintenance of a wide range of pathologies, including but not limited to anxiety and pain (47, 49–51).

**Figure 1:**
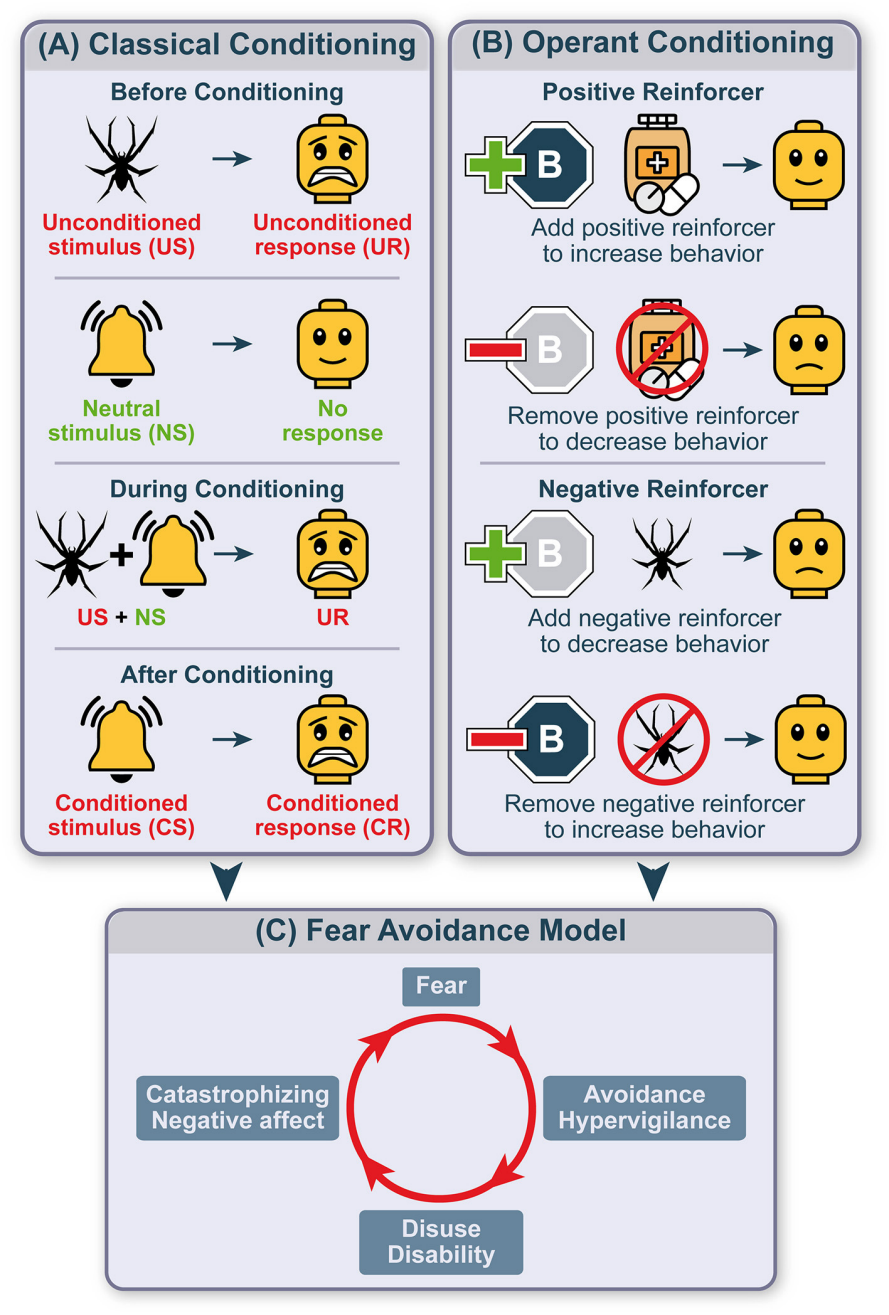
Avoidance within the conceptual framework of learning theory. Schematic depiction of avoidance embedded within the conceptual framework of classical conditioning initiating a fear response and mechanisms of operant conditioning fueling avoidance and maintaining maladaptive behaviors. Classical conditioning **(A)** refers to learning of an association between two stimuli. The unconditioned stimulus (US) naturally and automatically evokes an unconditioned response (UR) while neutral stimuli (NS) usually evoke no response. When associating the unconditioned with the neutral stimulus, the individual will display the unconditioned response. This association will turn the neutral into a conditioned stimulus (CS) evoking a conditioned response (CR). After a CS-US relationship is established, the behavior of an individual will be adapted based on its consequences. This operant conditioning **(B)** is formed by adding or removing a reinforcing stimulus that increases or decreases the probability of a specific behavior to occur in the future. A positive reinforcer increases the probability to show a certain behavior while its withdrawal discourages it. Adding a negative reinforcer decreases behavior while removing the negative reinforcer encourages the behavior to be displayed. These two processes combine to form a vicious circle of fear avoidance **(C)**. Upon the encounter of a fear-relevant stimulus, a vicious circle of fear avoidance is initiated by increasing attentional resources to potential sources of threat (hypervigilance), disuse and disability and exaggerating negative thoughts and affect (catastrophizing), thereby preventing the encounter with the feared stimulus and corrective measures.

Classical fear conditioning (Figure 1A) occurs through learning about the association between a neutral stimulus and an aversive event, e.g., a symptom, the unconditioned stimulus (US), which inherently elicits a defensive response (unconditioned response; UR), such as fear. Once established, this association renders the initially neutral stimulus a conditioned stimulus (CS), which now has acquired emotional value itself and is capable to trigger a response resembling the UR, the conditioned response (CR) (52, 53). According to the FAM, conditioned fear promotes multifaceted cognitive, emotional, and behavioral responses, including protective safety and avoidance behaviors. Avoidance and its short-term consequences, however, can initiate a second crucial learning process assumed to contribute to the transition from adaptive behavior to pathology, namely operant conditioning (54).

Operant conditioning or instrumental learning involves positive and negative reinforcers impacting the probability to execute behaviors based on their previous consequences (Figure 1B) (55–57). The presence of a positive reinforcer motivates the maintenance of a shown behavior, its absence, or removal reduces the probability to show this behavior in the future. Reversely, the occurrence of a negative reinforcer punishes performed behavior, resulting in its reduction. Finally, if a negative reinforcer is removed following a certain behavior, the behavioral strategy is strengthened. This last mechanism is considered key for the development and persistence of avoidance behaviors. Specifically, avoidance can instantly provide relief from states of fear, stress, and negative affect as significant negative reinforcers, and is thereby retained and even increasingly used as a behavioral response (46). Avoidance can be further used as a source of information, asserting that if avoidance was beneficial and relieving there must have been danger, and may thereby feed the original fear. Consequently, fear may prevail, resulting in the perpetuation of maladaptive avoidance, increased attention to threatening stimuli (hypervigilance), disuse and disability, catastrophizing, and negative affectivity (Figure 1C).

This two-stage theory of classical and operant conditioning, despite conceptual criticism (6), has been applied to panic disorder (58, 59), post-traumatic stress disorder (37, 60–62), phobias (63–65), social anxiety (66, 67), and chronic pain (68, 69). Common to all these conditions, sustained maladaptive avoidance behaviors prevent the individual from experiencing corrective situations that could rededicate a feared stimulus, such as a bodily symptom, as non-threatening, and can thus hamper fear extinction.

Extinction learning allows the formation of a new memory trace that inhibits the expression of previously acquired CS-US associations (70, 71). Exposure therapy follows the basic principles of extinction to support the development of new adaptive emotional, cognitive, and behavioral responses (28, 30). Importantly, extinguished threat responses may return under certain circumstances, such as with mere passage of time (“spontaneous recovery”), a context change (“renewal effect”), or an unexpected confrontation with the US (“reinstatement”). These return of fear phenomena may underlie relapse, posing major challenges to CBT (72, 73). As safety and avoidance behaviors during exposure therapy might compromise extinction learning (74) and can persist following experimental extinction training (75), they could also increase the risk of return of fear after successful exposure-based treatments (76).

It is therefore crucial to advance our understanding of avoidance behaviors, their underlying mechanisms, and their role in extinction-based treatments to both refine theoretical models and optimize therapeutic interventions for anxiety and psychosomatic disorders.

## Clinical Implications of Avoidance in Psychosomatic Medicine

The principles of learning theory and the key role of avoidance are increasingly recognized within biopsychosocial disease models and have been successfully translated into extinction-based interventions in various psychiatric and psychosomatic conditions. Inspired by their wide application in anxiety disorders (30, 32, 36, 77), exposure therapy has been established as an integral part of multimodal interventions for disorders characterized by disturbed interoception (78), including chronic pain (38, 54, 79, 80), disorders of the gut-brain axis, particularly irritable bowel syndrome (IBS) (48, 81–84), but also for body image disturbances and eating disorders (85–88), substance abuse and addiction (89–91).

Evidence supports the association between elevated levels of avoidance and pathology in these conditions (27, 41, 48, 78, 82), rendering avoidance a cardinal symptom and major target of CBT. In contrast to experimental settings, avoidance and safety behaviors in clinical populations are much more complex and highly patient- and disease-specific, ranging from refraining to enter a basement in spider phobia over lifting a suitcase only with severe tension in chronic low back pain to avoiding food consumption before a train ride in IBS. Therefore, it is crucial to individually tailor exposure therapy, which is often graded according to the patient's own threat hierarchy (48, 82).

From a clinical perspective, it is important to distinguish avoidance from safety behaviors and to adapt respective therapeutic approaches (2, 46, 92, 93). Safety behavior describes the endurance of a threat only when strategies aiming to prevent harm are simultaneously executed, such as calming self-talk, while avoidance aims at preventing the occurrence of the threatening stimulus itself, e.g., abstaining from feared situations, and eliminating a confrontation altogether. Accordingly, if avoidance is the disorder's maintaining factor, patients are encouraged to confront themselves with and approach the feared stimulus, optimally in different contexts to foster extinction generalization. To reduce safety behaviors, however, therapy should rather focus on the patient's strategies when being confronted with the feared object or situation.

In this manner, exposure therapy can demonstrably directly target maladaptive avoidance and safety behaviors in anxiety- and stress-related psychosomatic disorders (29, 40, 48, 82, 94, 95), which maintain symptom-related anxiety and contribute to symptom severity. Meanwhile, it is a matter of current debate whether adaptive and maladaptive use of avoidance behaviors should be dissociated, which may impact upon treatment outcome (96, 97). Specifically, avoidance behavior is often discouraged during exposure as it is considered to prevent a violation of dysfunctional expectancies (34, 35), thereby interfering with fear reduction and consequently maintaining symptoms or resulting in a return of fear (98). However, accumulating evidence suggests that avoidance does not necessarily hamper exposure therapy (99–101), may even facilitate its efficacy (102–104), and can enhance treatment acceptability and tolerability (105, 106). Therefore, understanding the process dynamics and considering adaptive characteristics of avoidance and safety behaviors appears crucial, as it may help to understand interindividual differences in risk of relapse.

## Challenges in Experimental Research on Avoidance

Despite the crucial impact of avoidance and safety behaviors in the development and maintenance of symptoms and the efficacy of therapeutic approaches, avoidance has long been widely neglected in experimental research (39), particularly in psychosomatic disorders. Recent advances have sparked renewed interest in the behavioral, neuroscientific, and clinical aspects of avoidance (2, 107, 108). However, several novel approaches have provoked criticism regarding validity criteria and translation into clinical reality (27, 44–47).

Commonly utilized experimental models often do not sufficiently represent the complex and nuanced dimensions of avoidance behaviors and their underlying learning processes in clinical populations. Emerging research has started to tackle this challenge with multifaceted translational approaches. Innovative examples range from the implementation of clinically-relevant, interoceptive visceral pain during associative learning (20, 109–111), over the use of immersive and interactive exposure techniques through virtual reality in phobias, anxiety disorders, and PTSD (112, 113), to a variation of the effort required to avoid movements using a robotic arm to address pain-related avoidance (114–116).

Likewise, a major pitfall in experimental avoidance research in humans is its operationalization as a dichotomous rather than continuous variable. In contrast to animal studies allowing a comprehensive evaluation of avoidance from subtle to excessive behaviors (117, 118), this complex phenomenon is often reduced to simple button presses indicating an evaluative decision to avoid or not to avoid an imminent outcome in human research (46). Attempts to overcome this lack of face validity have been made by including behavioral measures such as eye (119) and motion tracking (120) or by implementing a gradual admission of aversive outcomes (121).

Avoidance and safety behaviors can instantly relieve fear and distress and prevent the experience of an expected threat. As such, they serve as short-term rewards, motivating future performance. However, in pathology, excessive avoidance often results in a loss of long-term rewarding experiences, such as the engagement in social interactions, physical, or recreative activities. These avoidance costs contributing to deficits in quality of life are widely ignored in experimental research (118). One reason is likely the challenge to operationalize clinically-relevant costs in experimental settings. Avoidance costs, if considered, often involve instant monetary gains and losses (122–125). These approaches show low phenomenological validity, as they do not relate to the disease under investigation, cannot capture long-term costs of avoidance, and do not take interindividual variability in the value of costs into account. Innovative novel approaches, however, have been promising to address some of these concerns, incorporating the effort the individual is willing to put into avoiding an aversive US or targeting the relevance of avoidance costs in experimental approaches (114, 115, 126).

An even broader challenge is to index avoidance behaviors in both experimental and clinical settings alike. A patient with interoceptive visceral pain may perform a feared activity—yet does this with massive tension and hypervigilance, which can be difficult to identify and quantify as maladaptive behavior. There are various questionnaires and Behavioral Approach Tests (BATs) to quantify avoidance in clinical research and to evaluate the progress of behavioral treatments. However, several methodological weaknesses limit the psychometric and clinical utilization of these assessments (127), as they may partly depend on the instructions used and are prone to demand effects (128, 129). Likewise, available questionnaires in the context of chronic pain were often developed prior to the conceptual FAM and therefore lack construct validity, relevant cut-off scores, and responsiveness to treatment progress (130, 131).

Finally, avoidance is an instinctive survival behavior in response to environmental threats (132). Translating this evolutionarily hardwired protective response driven by severe fear of harm into experimental settings in human research has tight ethical boundaries. Most studies rely on instructed avoidance behavior and likely fail to capture the core mechanisms underlying avoidance as overt behavior (75, 133, 134). To target this issue, pain research addressed costly pain-related fear and avoidance more directly by implementing operant learning paradigms (115, 135, 136), documenting that sustained avoidance behavior is continued despite being no longer adaptive, and can even increase fear and pain sensitivity (137). It further underscores a key role of threat-related uncertainty, which has recently been identified as a putative vulnerability factor for maladaptive avoidance behavior (138), and may constitute a promising target for behavioral treatments in psychosomatic disorders (115).

From theoretical and clinical perspectives alike, expanding research on avoidance behavior is of key relevance to further elucidate the mechanisms and clinical implications of classical and operant learning, extinction, and the return of fear in psychosomatic disorders.

## Chances and Future Directions

To advance future research, it is crucial to validly address dysfunctional avoidance behavior and to foster a reciprocal translation between basic and clinical research through clinically-relevant experimental models. These should integrate behavioral approaches based on associative learning with cognitive theories of avoidance beliefs and schemas (139, 140). Not least in light of a need for individually-tailored CBT in anxiety- and stress-related disorders, it is key to acknowledge interindividual variability in aversive learning and memory, warranting more insight into putative moderating and mediating factors, and approaching predictions of specific avoidance patterns (6, 141).

For example, sex differences play an important role with women being more likely to engage in avoidance behaviors than men following traumatic events (142), in agoraphobia (143, 144), and in healthy individuals encountering panic-relevant (145) and phobic situations (146). Likewise, healthy women demonstrate longer avoidance duration and continue avoidant behavior during extinction (147), well in line with clinical data showing a higher female prevalence for anxiety disorders (148, 149), chronic pain (150), and IBS (151). Further, individual personality traits seem to influence the propensity to display maladaptive avoidance behavior. For example, evidence supports a relationship between intolerance of uncertainty, as well as neuroticism and avoidance (138, 152), being linked to higher fear avoidance both in healthy volunteers (153) and in clinical populations (154).

Experimental research further suggests that competing goals and goal prioritization in favor of reward seeking rather than threat avoidance attenuates the tendency to engage in avoidance behaviors (124, 155). Psychosomatic disorders are characterized by approach-avoidance conflicts following a continuum between the subjectively perceived threat and associated costs (156). While particularly anxious individuals tend to avoid a feared stimulus, associating the feared stimulus with higher rewards or avoidance costs may foster approach motivation (157) and promote fear extinction (158). Before a therapeutic intervention can be successful, it therefore appears crucial to evaluate and systematically implement what is rewarding to the individual to terminate dysfunctional and motivate more functional behaviors and non-avoidant decisions, which are demonstrably associated with less avoidance behavior post-treatment (123, 159).

Following theoretical accounts of learning, avoidance behaviors acquire inhibitory properties and are therefore presumed to interfere with fear extinction (76). Besides the need for further empirical testing of these assumptions (41, 160), it appears promising to target the extinction of maladaptive avoidance behaviors in CBT independent of the conditioned fear association (33, 161), particularly in patients who show excessive avoidance and those at risk to discontinue treatment. First experimental studies demonstrate that the extinction of avoidance behavior is facilitated by reducing the partial reinforcement rate of avoidance (133) and by increasing the effort (114, 158) or costs of an avoidance response (75, 162). These approaches may reduce the likelihood to express maladaptive avoidance behavior during exposure, paving the way for the successful extinction of conditioned threat associations.

Finally, approaches in experimental and clinical research alike should aim at bridging the gap between models applied in laboratory settings and patients' clinical reality and to more closely integrate the concepts of the FAM into the broad field of psychosomatic medicine. To achieve this goal, clinically-relevant and phenomenologically valid models are needed, capturing different facets as well as the specificity of fear and avoidance in psychosomatic disease, as first innovative attempts in the fields of muscoskeletal (115, 135–137, 163) and interoceptive visceral pain (20, 110, 111, 164, 165) have previously demonstrated. These experimental settings provide an ideal opportunity to overcome some common limitations of avoidance research, and to operationalize and assess the complex phenomenon of avoidance in its multiple facets, incorporating behavioral, cognitive, but also neural levels (39). Rather than artificial losses, clinically-relevant avoidance costs appear promising here, such as the previously implemented increased efforts to achieve a goal (135, 158) or a loss of predictability, which increases fear and uncertainty and can demonstrably affect pain-related fear and interoceptive pain experiences (111, 164, 166).

Albeit selective, this brief overview highlights key factors of relevance in experimental and clinical avoidance research in the context of anxiety- and stress-related disease. Future work in the field could, inspired and governed by associative learning principles, help shed light on mechanisms underlying different facets of adaptive and maladaptive avoidance as “the good, the bad, and the ugly” in pathology and therapy and pave the way toward refined tailored treatments for patients with psychosomatic disorders.

## Author Contributions

MW, SE, and AI acquired funding. FL, AI, and MW wrote the manuscript with input from all authors. SE made significant contributions to all parts of the manuscript. All authors approved the final version of the manuscript.

## Funding

This work was funded by the German Research Foundation (Deutsche Forschungsgemeinschaft, DFG), SFB 1280 *Extinction Learning* (316803389—Projects A10, A12, and A13).

## Conflict of Interest

The authors declare that the research was conducted in the absence of any commercial or financial relationships that could be construed as a potential conflict of interest.

## Publisher's Note

All claims expressed in this article are solely those of the authors and do not necessarily represent those of their affiliated organizations, or those of the publisher, the editors and the reviewers. Any product that may be evaluated in this article, or claim that may be made by its manufacturer, is not guaranteed or endorsed by the publisher.
